# Galvanic vestibular stimulation and its applications: a systematic review^☆^^☆☆^

**DOI:** 10.1016/j.bjorl.2022.05.010

**Published:** 2022-07-05

**Authors:** Anna Paula Batista de Ávila Pires, Tatiana Rocha Silva, Maíra Soares Torres, Maria Luiza Diniz, Maurício Campelo Tavares, Denise Utsch Gonçalves

**Affiliations:** a Universidade Federal de Minas Gerais (UFMG), Programa de Pós-Graduação em Ciências Fonoaudiológicas, Belo Horizonte, MG, Brazil; b Universidade Federal de Minas Gerais (UFMG), Programa de Pós-Graduação em Ciências da Saúde – Infectologia e Medicina Tropical, Belo Horizonte, MG, Brazil; c Universidade Federal de Minas Gerais (UFMG), Programa de Residência Médica, Faculdade de Medicina, Belo Horizonte, MG, Brazil; d Universidade Federal de Minas Gerais (UFMG), Faculdade de Medicina, Belo Horizonte, MG, Brazil; e Contronic Sistemas Automáticos Ltda, PDI – Pesquisa, Desenvolvimento & Inovação, Pelotas, RS, Brazil; f Universidade Federal de Minas Gerais (UFMG), Faculdade de Medicina, Departamento de Otorrinolaringologia, Belo Horizonte, MG, Brazil

**Keywords:** Postural balance, Central nervous system diseases, Vestibular diseases, Electric stimulation, Cognition

## Abstract

**Objective:**

Galvanic vestibular stimulation has been evaluated in the context of vestibular rehabilitation. The objective was to identify evidence in the scientific literature about the clinical applications of galvanic vestibular stimulation.

**Methods:**

In this systematic review, the articles describing the applications of galvanic vestibular stimulation were extracted from PubMed, Web of Science, MEDLINE, Scopus, LILACS and SciELO databases. The survey was limited to articles published in English, Portuguese and Spanish. All the articles about the clinical applications of galvanic vestibular stimulation were compiled. Repeated articles in the databases, literature review articles, case reports, letters and editorials were excluded. The descriptors included: galvanic vestibular stimulation, postural balance, central nervous system diseases, vestibular diseases, spinal cord diseases and cognition.

**Results:**

The search strategy resulted in the initial selection of 994 articles; the reading of titles and abstracts was accomplished in 470 articles and the complete reading in 23 articles. Clinical applications of galvanic vestibular stimulation included Ménière’s disease, vestibular neuritis, bilateral vestibular disorders, vestibular schwannoma, Parkinson’s disease, ischemic central lesions, motor myelopathies, anxiety disorders, cognition and memory.

**Conclusion:**

Galvanic vestibular stimulation has been considered a potentially useful strategy for balance rehabilitation, since it has the effect of stimulating the central connections related to the postural balance, favoring new neuronal synapses that allow the partial or total recovery of postural imbalance.

## Highlights

• Galvanic vestibular stimulation is used for peripheral and central vestibular rehabilitation.
• Galvanic vestibular stimulation partially or totally improves body balance.
• Galvanic vestibular stimulation has also therapeutic application in cognition and mood.

## Introduction

Galvanic vestibular stimulation (GVS) was discovered in the early 19th century.^1^, ^2^ GVS is a non-invasive method used to stimulate the vestibular system, including vestibular sensors, neural pathways, vestibular nuclei, and cortical areas that receive integrated vestibular inputs.^3^

GVS involves transcranial stimulation by a direct current, which both, stimulates and inhibits vestibular afferents.^4^, ^5^ The vestibular nuclei are polarized, which means that the GVS separates and accumulates positive (cathode) and negative (anode) electrical charges at distinct regions, creating a dipole between the vestibular nuclei. This process activates the semicircular canals, otolith organs, and adjacent vestibular nerves.^6^, ^7^ Thus, the GVS modulates posture and balance,^8^, ^9^, ^10^ oculomotor responses,^11^, ^12^ and spatial orientation.^13^

Electrical stimulation is conducted towards the vestibular nerve and then to the vestibular nuclei in the brainstem which, in turn, are interconnected with the thalamic relay stations (ventral posterolateral nucleus). From this point, the vestibular ascending pathways will synapse on vestibular cortical areas, including the central sulcus, somatosensory cortex, parietal area, and insular parietal vestibular cortex.^14^ As for the descending pathways, the stimulus reaches the vestibulospinal and reticulospinal tracts in the spinal cord, generating a postural response.^12^ Some authors consider that GVS acts on all pathways involved in the conduction of a vestibular reaction along the spinal cord, including the vestibular, reticular and corticospinal tracts.^15^

Stimulation of the lateral vestibular nucleus in its ventral portion acts on the vestibulo-ocular circuits through afferents to the utricle and semicircular canals. The stimulation of the dorsal portion of this nucleus excites projections, via the lateral vestibulospinal tract, which have an effect on the motoneurons that innervate the muscles of the lower limbs, to cause tonic excitation in the leg extensor muscles, contributing to the maintenance of posture.^14^

Stimulation of the inferior vestibular nucleus excites afferents from the semicircular canals, saccule and utricle, in addition to cerebellar projections. Its projections include vestibulospinal circuits, integrating vestibular and cerebellar afferents.^14^, ^15^, ^16^

As for the GVS technique, surface electrodes are fixed on the mastoids and the electrical stimulus is applied, being generally characterized by a pulsed direct current of low amperage, with a cathode on one mastoid and an anode on the other. This electrical dipole generates stimulation of vestibular afferents on one side while, at the same time, generates contralateral inhibition. Rapid alternation in the electrical dipole may favor a vestibular rehabilitation process, in which the cortical projections that constitute the vestibular cortex are modulated for a better postural response.^17^, ^18^, ^19^ Although the stimulators used to generate the GVS are essentially similar, the changes in body perception, movement, and spatial location that the GVS produces are based on wave configuration, polarity, intensity, duration, time, and frequency of stimulation.^20^, ^21^

Changes in the vestibular input (cathode or anode) exert a strong influence on the subject’s posture^22^, ^23^, ^24^ and standing balance.^25^ In addition to its role in gaze stabilization and postural control, the vestibular system is involved in some cognitive functions and emotional processing.^26^, ^27^ Several studies have disclosed a modulating effect of vestibular stimulation on mood, emotional control, and level of anxiety.^28^, ^29^, ^30^

Currently, GVS has been used as a diagnostic and rehabilitative resource in vestibular disorders, such as vestibular neuritis,^31^ Ménière’s disease,^32^ bilateral vestibular disorders,^32^, ^33^, ^34^, ^35^ and vestibular schwannoma.^36^ Among the central diseases, Parkinson's disease,^37^, ^38^, ^39^, ^40^, ^41^, ^42^ central ischemic lesions^43^, ^44^ and motor myelopathies stand out.^45^ GVS is also applied in anxiety disorders^46^ and to improve cognition^47^ and memory.^48^, ^49^ This advance in research on the use of GVS in clinical practice stems from favorable characteristics for its usage, such as objectivity, safety, easy performance, low cost, fastness and minimal discomfort for the patient.

The main objective of this systematic review is to verify the scientific evidence on the clinical applications of GVS.

## Methods

This systematic review sought to answer the following question: “what are the applications of galvanic vestibular stimulation for diagnosis and rehabilitation?”. The search strategy was based on the acronym PICO, which represents the four fundamental components of question construction for the bibliographic search in the research: Patient, Intervention, Comparison and Outcome. This systematic review was performed according to the Preferred Reporting Items for Systematic Reviews and Meta-Analyses Statement (PRISMA) recommendation.^50^ The protocol was registered in August 2021 in the International Prospective Register of Systematic Reviews – Prospero (https://www.crd.york.ac.uk/PROSPERO/) database under registration number CRD272303.

### Search strategy

The descriptors comprised “postural balance”, “central nervous system diseases”, “vestibular diseases”, “spinal cord diseases” and “cognition”, and the free term included was “galvanic vestibular stimulation”. The descriptors were selected based on the consultation of DeCS (Descriptors in Health Sciences) and MeSH (Medical Subject Headings) and were combined with the free term, using the Boolean operator AND. The following combinations were used: “galvanic vestibular stimulation AND postural balance”, “galvanic vestibular stimulation AND central nervous system diseases”, “galvanic vestibular stimulation AND vestibular diseases”, “galvanic vestibular stimulation AND spinal cord diseases” and “galvanic vestibular stimulation AND cognition”. There was no restriction on the language of the publication.

The search was conducted in July 2021 in the electronic databases PubMed, Web of Science, MEDLINE, Scopus, LILACS and SciELO. After the search, the references of each database were exported to the Mendeley® program (https://www.mendeley.com/) aiming to identify all duplicate articles, promote greater selection reliability and continue onto the article eligibility stage.

### Eligibility criteria

The articles that met the following criteria were included in this review: (1) Publications in Portuguese, English or Spanish; (2) The title should contain the word GVS and the clinical application should be included in the title or abstract. Articles that did not mention the characteristics of the used GVS or did not describe the results of the GVS were excluded. Repeated articles in the databases, literature review articles, case reports, letters and editorials were also excluded.

### Data analysis

For the analysis of the selected articles, the “Strengthening the Reporting of Observational Studies in Epidemiology (STROBE)” recommendations were used.^51^ In the article selection process, after the initial exclusion of articles that were outside the scope of this review, the analysis continued by reading the titles and abstracts of the remaining articles. Those articles that met the inclusion criteria and did not meet the exclusion criteria were read in full. After reading and analyzing these articles, the selected information was: authors, year of publication, country where the research was developed, characterization of the method, number of subjects, clinical application and study results. A descriptive analysis of the results was carried out and, due to the heterogeneity of the data and methodology, it was not possible to carry out a meta-analysis.

## Results

Using the search strategies, a total of 994 publications were identified (349 in PubMed, 275 in Web of Science, 199 in MEDLINE, 167 in Scopus and 4 in the SciELO databases). There were no publications in the LILACS database.

**Figure 1:**
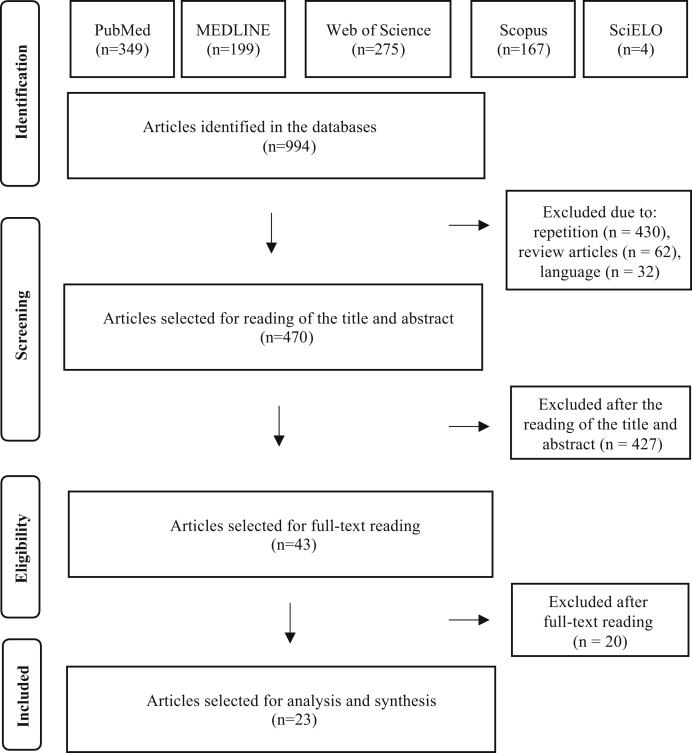
Identification and selection flow diagram.

After eliminating 430 studies in duplicate, 62 review articles and 32 studies in languages other than the three admitted ones, 470 articles were selected for the reading of titles and abstracts. After reading the titles and abstracts of these articles, 427 studies were excluded, according to the established selection criteria, and 43 articles were selected for reading in full. After the reading, 20 articles were excluded because they did not contain data on GVS. Finally, 23 full articles were included in the qualitative analysis. The entire article selection process is described in Fig. 1, which shows the PRISMA flow diagram for inclusion.

Table 1 shows a summary of the 23 studies included in the review.

**Table 1:** Characterization of the 23 studies selected for systematic review.

Author	Year/location		Design	Population	Characterization of GVS	Application
Vailleau et al.^31^	2011/Paris (France)		Comparative cross-sectional	42 individuals with vestibular neuritis and Ménière disease	Intensity: 4 mA	GVS to diagnose residual vestibular function
				and control group	Freq.: 50 Hz	
Ko et al.^32^	2020/Taipei (Taiwan)		Comparative cross-sectional	7 individuals with bilateral vestibular hypofunction and control group	Intensity: from 200 to 1000 μA	To evaluate the effects of GVS on postural instability improvement
					Freq.: 100 Hz	
Fujimoto et al.^33^	2018/Tokyo (Japan)		Descriptive cross-sectional	13 individuals with bilateral vestibulopathy	Intensity: 100 to 1000 μA	To evaluate the effects of GVS on postural instability improvement
					Freq.: 20 Hz	
Fujimoto et al.^34^	2016/Tokyo (Japan)		Comparative cross-sectional	30 healthy elderly divided into 2 groups	Intensity: from 0.5 to 2 mA	To evaluate the effects of GVS on postural instability improvement
					Freq.: from 1 to 10 Hz	
Sprenger et al.^35^	2020/Lüebeck (Germany)		Comparative cross-sectional	30 individuals with bilateral vestibular hyporeflexia and control group	Intensity: 0.5 to 1.5 mA	GVS as a predictor of postural control safety and risk of fall
					Freq.: not informed	
Welgampola et al.^36^	2013/London (England)		Comparative cross-sectional	10 individuals with vestibular Schwannoma and control group	Intensity: 1 mA	GVS to diagnose residual vestibular function
					Freq.: 200 Hz	
Liu et al.^37^	2021/Hefei (China)		Comparative cross-sectional	27 individuals with Parkinson’s disease and control group	Intensity: not informed	To evaluate the effects of GVS on the improvement of motor deficits, balance and interhemispheric connectivity deficiency
					Freq.: from 70 to 100 Hz	
Cai et al.^38^	2018/Vancouver (Canada)		Comparative cross-sectional	23 individuals with Parkinson’s disease and control group	Intensity: not reported	GVS to increase Pedunculopontine nucleus Connectivity
					Freq.: 128 Hz	
Kataoka et al.^39^	2015/Nara (Japan)		Descriptive cross-section	5 individuals with	Intensity: 0.7 mA	To evaluate the effects of GVS on postural instability improvement
				Parkinson’s disease	Freq.: 100 Hz	
Pal et al.^40^	2009/Sydney (Australia)		Comparative cross-sectional	5 individuals with Parkinson’s disease and control group	Intensity: 0.1 to 0.5 mA	GVS to reduce balance in postural assessment
					Freq.: 200 Hz	
Khoshnam et al.^41^	2018/Canada	Descriptive cross-sectional		11 individuals with Parkinson’s disease	Intensity: 10 μA	To evaluate the effects of GVS on motor symptoms of upper and lower limbs
					Freq.: not informed	
Okada et al.^42^	2015/Koryo-cho (Japan)	Longitudinal clinical trial		7 individuals with Parkinson’s disease and control group	Intensity: 0.7 mA	To evaluate the effects of GVS on the anterior flexion angle
					Freq.: not informed	
Oppenländer et al.^43^	2015/Saarbruecken (Germany)	Longitudinal cohort		24 individuals with CVA and non-exposed group	Intensity: 0.7 mA	To evaluate the effects of GVS on post-CVA verticality deficits in the visual and tactile modality
					Freq.: not informed	
Saj et al.^44^	2005/Lille (France)	Comparative cross-sectional		17 individuals with hemispheric lesion and control group	Intensity: 1.5 mA	Investigate the effects of GVS on subjective vertical vision
					Freq.: not informed	
Čobeljić et al.^45^	2018/Belgrade (Serbia)	Descriptive cross-sectional		7 individuals with complete spinal cord injury	Intensity: 4 mA	GVS to determine changes in clinical and biomechanical measures of spasticity
					Freq.: not reported	
					Time: 15 s	
Pasquier et al.^46^	2019/Caen (France)	Descriptive cross-sectional		22 individuals with a history of anxiety	Intensity: 1 mA	To evaluate the tolerability and efficacy of GVS in the treatment of anxiety
					Freq.: not informed	
Dilda et al.^47^	2012/New York (United States)	Descriptive cross-sectional		120 healthy subjects	Intensity: from 1 to 5 mA	To evaluate the effects of GVS on cognition
					Frequency: from 0.16 to 0.61 Hz	
Hilliard et al.^48^	2019/Dresden (Germany)	Descriptive cross-sectional		47 healthy subjects	Intensity: from 0.25 to 1.25 mA	To evaluate the effects of GVS on spatial learning and memory
					Freq.: from 0.1 to 100 Hz	
Wilkinson et al.^49^	2008/Boston (United States)	Comparative cross-sectional		24 healthy individuals, divided into 2 groups	Intensity: 3 mA	To evaluate the effects of GVS on visual memory
					Freq.: 1000 Hz	
Ceylan et al.^52^	2020/Istanbul (Turkey)	Comparative cross-sectional		42 subjects complaining of vertigo lasting longer that 1 year and control group	Intensity: from 1 to 5 mA	GVS to promote improvement in vestibular rehabilitation
					Freq.: 100 Hz	
Nooristani et al.^53^	2019/Montreal (Canada)	Comparative cross-sectional		28 young adults divided in 2 groups	Intensity: 1 mA	To evaluate the effects of GVS on postural improvement
					Freq.: from 0 to 640 Hz	
Inukai et al.^54^	2018/Niigata (Japan)	Longitudinal clinical trial		32 elderly and control group	Intensity: 0.4 mA	To evaluate the effects of GVS on postural instability improvement
					Frequency: from 0.1 to 640 Hz	
Carmona et al.^55^	2011/Rosario (Argentina)	Longitudinal clinical trial		19 subjects with uncompensated unilateral peripheral vestibular syndrome and control group	Intensity: 0.5 to 2 mA	To evaluate the long-term effects of GVS on temporal sway improvement
					Freq.: 0 to 1 Hz	

GVS, Galvanic vestibular stimulation; Ma, milliampere; μA, microampere; Hz, Hertz; CVA, cerebrovascular accident.

The variables language, country of origin and study design were described to help characterize the studies included in the review, but they are not part of the main outcomes.

All 23 selected articles were published in English, between 2005 and 2021. The countries with the highest number of publications were: Japan with 5 (23%) publications^33^, ^34^, ^39^, ^42^^,^^54^ and France,^31^, ^44^, ^46^ Germany^35^, ^43^, ^48^ and Canada,^38^, ^41^, ^53^ with 3 each (14%). The sample size of the studies ranged from 5 to 120 individuals with peripheral and central vestibular alterations.

As for the design, 7 (30%) studies were descriptive,^33^, ^39^, ^41^, ^45^^,^^46^, ^48^ 12 (53%) were comparative cross-sectional studies^31^, ^32^, ^34^, ^35^, ^36^, ^37^, ^38^, ^40^, ^44^, ^49^^,^^52^, ^53^ and 3 (17%) studies were longitudinal.^42^, ^43^, ^54^, ^55^

Regarding the population/sample of individuals included in the studies, the most often investigated clinical applications were related to Parkinson’s Disease,^37^, ^38^, ^39^, ^40^, ^41^, ^42^ bilateral vestibular disorders,^32^, ^33^, ^35^ and central diseases.^43^, ^44^, ^45^

In the 23 articles analyzed, the GVS was used for the most diverse purposes. GVS for vestibular function rehabilitation was the most frequently used application.^32^, ^33^, ^34^, ^35^, ^37^, ^39^, ^40^^,^^52^, ^53^, ^54^, ^55^

Regarding the characterization of the GVS, it was observed that the current and frequency were variable^31^, ^32^, ^33^, ^34^, ^36^, ^39^, ^40^^,^^47^, ^48^, ^49^, ^52^, ^53^, ^54^, ^55^ and some studies^35^, ^37^, ^38^, ^39^, ^41^, ^42^, ^43^, ^44^, ^45^, ^46^ did not disclose all the characteristics of the methods of the investigation.

## Discussion

The present review showed that GVS has a clinical application in Ménière’s disease, vestibular neuritis, bilateral vestibular disorders, vestibular schwannoma, Parkinson’s disease, central ischemic lesions, motor myelopathies, anxiety, cognition and memory disorders, and age-related instability.^32^, ^33^, ^34^, ^35^, ^37^, ^38^, ^39^, ^40^, ^41^, ^42^, ^43^, ^44^, ^45^, ^46^, ^47^, ^48^, ^49^, ^52^, ^53^, ^54^, ^55^ These applications are justified by the stimulating effect of GVS on the central nervous system, creating neuronal connections that allow partial or total recovery of the lost vestibular function and the connection between the vestibular pathways and the limbic system.

In healthy young subjects, changes were observed in the parameters of center of mass sway assessed in the posturography test after the use of GVS, although there were no significant changes compared to the placebo group.^53^

In healthy elderly subjects, the use of GVS improved postural instability assessed by the posturography test.^34^, ^54^ These elderly showed improvement in the parameters of center of mass sway, whose gain remained after a few hours of stimulation.

GVS can induce an improvement in postural stability after the end of the stimulus due to a strong post-stimulation effect. The repetition of the stimulus may induce further and sustained improvement.^34^ These effects may contribute to the greater applicability of GVS in postural stabilization in adults and the elderly.^34^

Cognitive aspects were also improved with the application of GVS, such as spatial learning, executive memory^48^ and visual memory.^47^, ^48^, ^49^

GVS can be used for diagnostic purposes. GVS, followed by assessment of the vestibulo-ocular reflex via videonystagmography was used in patients with peripheral vestibular hypofunction.^31^ GVS stimulates the residual vestibular function (e.g., patients with bilateral areflexia on caloric testing), and if any reflex ocular response is present based on the videonystagmography, this is an indication that the residual vestibular function is present.^31^

GVS was used in patients with vestibular schwannoma to assess the impact on body balance generated by GVS in relation to healthy controls. They concluded that the application of GVS associated with the measurement of body balance allows the assessment of the postural function of individuals with unilateral vestibular loss.^36^

GVS was effective in demonstrating vestibular response asymmetry.^36^ The intensity of the applied current was 1 mA, at a frequency of 200 Hz, for 3 s.

GVS has also shown to be useful in determining the level of spinal cord injury in patients with motor myelopathy.^56^ The applied current intensity was 2 mA, at a frequency of 1 Hz, for 400 milliseconds.

The most important application of GVS in terms of the potential use in clinical practice is for Vestibular rehabilitation (VR). GVS has been used in individuals with uncompensated unilateral vestibular hypofunction, bilateral vestibular hypofunction, and postural instabilities related to neurological diseases.^32^, ^33^, ^35^, ^37^, ^38^, ^39^, ^40^, ^42^, ^52^, ^55^

In uncompensated unilateral vestibular hypofunction, the results in terms of body balance gain were better with GVS associated with Cawthorne and Cooksey exercises (experimental group) compared to vestibular rehabilitation using only Cawthorne and Cooksey exercises (control group).^52^ Both groups underwent a six-week rehabilitation protocol. GVS was applied once a week for six weeks in the experimental group. Both groups were instructed to perform the exercises every day, five times a day. Weekly balance assessments were performed throughout the period and the results in terms of body balance gain in the experimental group were superior to the results obtained in the control group.^52^

In bilateral vestibular hypofunction, GVS was used to stimulate body balance in two sessions with a 14-day interval between them. The posturography parameters were evaluated immediately after stimulation and within six hours in both stimulation sessions. In both sessions, an improvement was observed in the posturography parameters related to the center of pressure with less sway, which was maintained for 6 h following the stimulus.^33^ The long-term beneficial effect remains unknown. Other studies confirmed that GVS was an effective strategy to improve body balance in patients with bilateral vestibular hypofunction.^32^, ^35^

The effects of GVS postural improvement on bilateral vestibular hypofunction seems to be smaller for tasks that require more demanding postural control conditions, such as soft surfaces and cognitive distractions, which are closer to everyday conditions.^35^ In the utilized protocols, the current intensity varied from 0.1 to 1.5 mA, with a frequency that varied between 20 Hz and 100 Hz. The stimulation time ranged from 6 to 30 min.^32^, ^33^, ^35^

GVS can improve postural instability due to neurological disorders.^37^, ^38^, ^39^, ^40^, ^42^ To date, the sites of GVS action on the central nervous system remain unclear.^37^, ^38^, ^39^, ^40^, ^41^, ^42^ It is known that instability of central causes shows little improvement with traditional VR methods.^37^, ^39^, ^40^ When comparing the GVS protocols used for the rehabilitation of instability of central causes with those used for peripheral causes, the intensity of the current was lower in the first. The authors did not report a reason for using the lower stimulus. An exception was observed for complete spinal cord injury. In this case, the GVS was used to assess the biomechanical changes in muscle spasticity generated by the spinal cord injury, and the intensity of the applied current was 4 mA, similar to the stimulus used in diseases with unilateral peripheral vestibular hypofunction.^31^, ^45^

In Parkinson’s disease, imbalance and falls are resistant to treatment with medications and surgical interventions. Neuroimaging studies suggest that the use of GVS may improve the connectivity deficiency present in the peduncle pontine nucleus in Parkinson’s disease.^38^ The use of GVS with current intensity varying between 0.1 and 0.7 mA promoted a small reduction in the body mass center sway in individuals with Parkinson’s disease in some situations, when compared to the control group.^37^, ^39^, ^40^ GVS seems to play a role in improving upper and lower-limb motor symptoms related to Parkinson’s disease.^41^

Studies evaluating the postural instability of Parkinson’s disease, through the pull test applied before and after stimulation, demonstrated that GVS can be a resource used to improve the postural instability of these patients.^42^ As a stimulation protocol, the current ranged from 0.01 mA to 0.7 mA, with a frequency between 70 and 200 Hz.^37^, ^38^, ^39^, ^40^, ^41^, ^42^ The stimulation time lasted 20–26 s in studies that evaluated the influence of stimulation in upper and lower-limb motor symptoms^40^, ^41^ and 2 min in studies that evaluated brain areas stimulated by GVS.^37^, ^38^ Protocols with stimulation duration of 20 min were used for postural rehabilitation purposes.^39^, ^42^

In central ischemic injuries, GVS significantly contributed to the recovery of visual and tactile verticality deficits after a stroke, highlighting the importance of the vestibular system in the multimodal subjective vertical perceptions.^43^, ^44^ In stroke and hemiparetic lesions, the current intensity ranged from 0.7 mA to 1.5 mA. The time of GVS application aiming at sensory deficit rehabilitation was 20 min, similar to the protocol used for the same purpose in Parkinson’s disease.^39^, ^42^, ^43^

In motor myelopathies, the use of GVS has been tested as a therapeutic resource in an attempt to reduce muscle spasm in individuals with complete spinal cord injury. Although the response was not statistically significant when compared to the placebo stimulus, some subjects showed improvement in objective tests.^45^ The stimulation protocol used a current of 4 mA for 15 s.^45^

In psychiatric conditions, GVS has also shown good results.^46^ In anxiety disorders, GVS was able to reduce anxiety symptoms without causing significant postural change or discomfort in individuals.^46^ The existence of a relationship between the vestibular stimulation pathway and the pathways related to anxiety, depression, and cognition is well established.^46^, ^47^ The current intensity used ranged from 0.25 mA to 3 mA, with stimulation frequency varying between 0.1 and 1000 Hz.^46^, ^47^, ^48^, ^49^

Regarding the technical parameters of GVS, the studies, whether for diagnosis or for vestibular rehabilitation, showed variable parameters in relation to the intensity of the used current, stimulation duration, number of repetitions and duration of treatment. The stimulus site, electrode type and current type did not vary. GVS was applied to the mastoid and surface electrodes were used in all studies. The type of used current was always the alternating type, which is safer for application in humans as it has a lower risk of tissue damage by heating and electrolyte dissociation.^57^ In protocols in which the GVS effect assessment was performed simultaneously with the stimulus application, the stimulation time varied from 10 to 20 s.^48^, ^49^ In the protocol in which the assessment was performed using scales applied before and after the stimulus, GVS application time was 15 min.^47^ The methodological differences related to the parameters time, intensity, stimulus frequency and treatment duration limited the comparison between the studies and prevented conducting a meta-analysis.

The effect of GVS on the synaptic circuits that organize vestibular reflexes is gradual.^3^, ^20^ There is evidence that the connections in these circuits exhibit a high degree of plasticity, involving rearrangements of the synaptic circuits that organize vestibular reflexes.^20^ It takes some time of GVS use for the activation of these neuroplasticity mechanisms. The literature has not yet defined how long the beneficial effect of GVS remains.

The small sample size is a limitation of studies evaluating the use of GVS for body balance rehabilitation.^31^, ^32^, ^33^, ^35^, ^39^, ^40^^,^^42^, ^52^, ^55^ The study with the largest sample size comprised 42 participants, which does not allow the study power control.^52^ On the other hand, GVS showed to be useful as a therapeutic resource in several diseases that affect the nervous system. Therefore, based on the reviewed studies, GVS has shown to be an option as a therapeutic resource to improve postural stability, cognition and mood.

It is important to note that we did not find any publications that show GVS as an ineffective method in VR. The lack of these studies reinforces the importance of well-controlled studies on GVS and with a larger sample size to be developed and published before introducing GVS into clinical practice. Non-significant results may not have been published, causing a bias in the analysis.

## Conclusion

Despite the limited sample of patients in the articles and the methodological differences that make it difficult to compare the results between the studies, GVS showed to be a safe, low-cost, easy to perform, non-invasive and effective tool for clinical application in vestibular rehabilitation.

## Conflicts of interest

The authors declare no conflicts of interest.
